# A Compact Review of IPMC as Soft Actuator and Sensor: Current Trends, Challenges, and Potential Solutions From Our Recent Work

**DOI:** 10.3389/frobt.2019.00129

**Published:** 2019-12-05

**Authors:** Muyu Hao, Yanjie Wang, Zicai Zhu, Qingsong He, Denglin Zhu, Minzhou Luo

**Affiliations:** ^1^School of Mechanical and Electrical Engineering, Hohai University, Changzhou, China; ^2^Jiangsu Key Laboratory of Special Robot Technology, Hohai University, Changzhou, China; ^3^Jiangsu Provincial Key Laboratory of Bionic Functional Materials, Nanjing University of Aeronautics and Astronautics, Nanjing, China; ^4^School of Mechanical Engineering, Xi'an Jiaotong University, Xi'an, China

**Keywords:** IPMC, relaxation effect, solvent evaporation, output force, soft robotics

## Abstract

Recently, attempts have been made to develop ionic polymer–metal composite (IPMC), which is garnering growing interest for ionic artificial muscle, as a soft actuator and sensor due to its inherent properties of low weight, flexibility, softness, and particularly, its efficient transformation of electrical energy into mechanical energy, with large bending strain response under a low activation voltage. In this paper, we focused on several current deficiencies of IPMC that restrict its application, such as non-standardized preparation steps, relaxation under DC voltage, solvent evaporation, and poor output force. Corresponding solutions to overcome the abovementioned problems have recently been proposed from our point of view and developed through our research. After a brief introduction to the working mechanism of IPMC, we here investigate the key factors that influence the actuating performance of IPMC. We also review the optimization strategies in IPMC actuation, including those for preparation steps, additive selection for a thick casting membrane, solvent substitutes, water content, encapsulation, etc. With consideration of the role of the interface electrode, its effects on the performance of IPMC are revealed based on our previous work. Finally, we also discuss IPMCs as potential sensors theoretically and experimentally. The elimination of the deficiencies of IPMC will promote its applications in soft robotics.

## Introduction

In recent years, with developments in the field of soft robotics, new demands have emerged that actuators and sensors for use in such robotics must be flexible and miniaturized. Ionic polymer–metal composites (IPMC) have been studied as promising smart materials for use as actuators and sensors in soft robotics and other industrial applications during the last two decades (Tiwari and Garcia, 2011; Bhandari et al., 2012; Kim, 2013). As a kind of electroactive polymer (EAP), IPMC has some distinct advantages for use in soft robotics. Its advantageous features include but are not limited to low weight, flexibility, softness, and especially, efficient transformation of electrical energy to mechanical energy, with a large bending strain response to low activation voltage (1~3 V), low power consumption, rapid response, mechanical and chemical tolerance and stability, ease of miniaturization, and so on. Due to the above characteristics, IPMC can be used as soft actuators in soft robotics, especially microrobots and underwater robots. Additionally, IPMC can also convert mechanical energy into electrical energy. Because of its self-sensing capacity, it has some favorable properties for use in the sensing field, as well.

**Figure 1:**
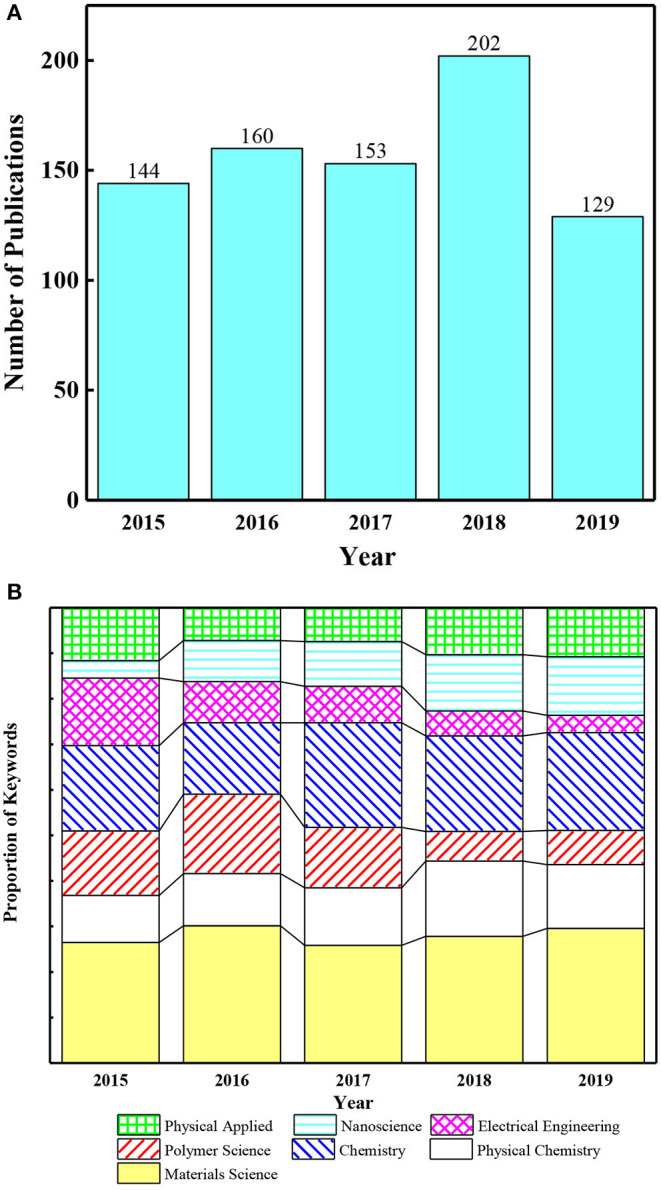
**(A)** Number of publications during the last 5 years (data from WOS to August 2019). **(B)** Recent research hotspots in IPMC.

Many potential uses for IPMC have already been proposed by researchers (Bonomo et al., 2005; Shahinpoor and Kim, 2005). Mojarrad used IPMC for the propeller of a biomimetic robotic underwater propulsion system (Mojarrad and Shahinpoor, 1997). A snake-like swimming robot using an IPMC actuator/sensor was proposed by Kamamichi et al. (2006). In 2007, Guo et al. used IPMC as part of the actuators developed for a jellyfish-like underwater microrobot (Guo et al., 2007). Krishen studied the application of IPMC for space missions (Krishen, 2009). In 2015, Shen et al. developed a biomimetic underwater vehicle with IPMC sensors (Shen et al., 2015). Palmre et al. fabricated a bio-inspired IPMC actuator as an active fin that was capable of bending and twisting (Palmre et al., 2012). As soft actuators, as well as for use in robotics, IPMCs also have applications in optical systems, such as an auto-focus compact camera module and a tilting actuator for an endoscope (Kim et al., 2012; Tsai et al., 2012). Additionally, Tripathi et al. developed an IPMC actuator that was used as a prototype for an active catheter-guidewire maneuvering application (Tripathi et al., 2019). In 2018, an IPMC-based refreshable braille display application was reported (Feng and Hou, 2018). Recently, Chang et al. designed a drug-release device using IPMC for biomedical applications (Chang X.L. et al., 2018). However, there still are some inherent challenges, such as relaxation under direct current (DC) voltage, poor output force, solvent evaporation, and non-standardized preparation steps, that restrict IPMC use in the sensing and actuating fields, and there is still a large amount of room for improvement (Jung et al., 2003; Lee et al., 2005). In recent years, to overcome these unfavorable factors, a series of solutions have been proposed for its further application in soft robotics. Optimizing and improving the preparation steps will make the surface electrode layers more homogeneous, so that a sample with uniform performance can be obtained. To overcome the problem of relaxation, some efforts had been made to adjust the water content of water-based IPMC or replace the water with other non-volatile solvents (such as ionic liquids), and so on. In the past 5 years, research regarding IPMC for soft robotics applications has aroused widespread interest in various fields, including materials science, physical chemistry, chemistry, polymer science, electrical engineering, nanoscience, and applied physics, as shown in Figure 1.

This paper intends to review and discuss the effects of these solutions on the relief of the inherent defects of IPMC, considering the optimization of the surface electrode, polymer membrane, electrode interface, and so on. This review consists of three sections as follows. After the working mechanism of IPMC has been introduced, the methods for optimizing IPMC actuating performance are discussed, including the optimization of the preparation steps, relaxation elimination, and output force enhancement. Finally, the use of IPMCs as sensors is briefly discussed.

## Working Mechanism of IPMC

**Figure 2:**
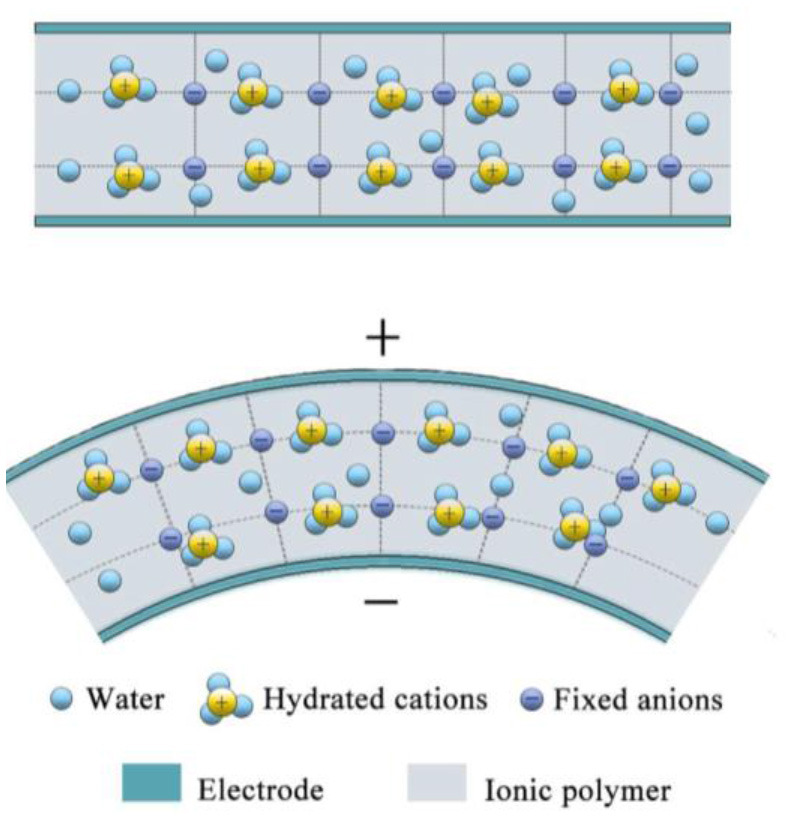
Ionic polymer–metal composite (IPMC) sensing under bending deformation (Wang et al., 2019) (reprinted with permission).

Generally, IPMC consists of three layers, specifically a thin electrolyte membrane with two noble metal electrode layers on either side, so that the structure of IPMC is like a sandwich. The actuating mechanism of IPMC is shown in Figure 2. Due to their similar chemical structures and properties, Nafion, Flemion, Aciplex, etc. are the most used materials for the base membrane of IPMC. A conductive electrode layer is plated on each side of the base membrane using noble metal salts or a mixture of such salts, including gold, platinum, palladium, etc. When an electric field is applied to the IPMC, the cations, together with water molecules, will move toward the cathode. Due to the anisotropic concentration distributions of cations and water molecules, strain will be generated near the cathode of the IPMC, and the IPMC will bend toward the anode (Shahinpoor, 2003; Schmidt-Rohr et al., 2008). Likewise, when mechanical deformation or another stimulus is applied to IPMC, ion migration will occur due to the strain gradient inside the IPMC and will then result in a potential difference on both sides of the IPMC. According to the above depiction, there are actually four components to constructing IPMC, namely the mid-layer, cations, solvents, and electrode layers. Many researchers have therefore devoted themselves to improving the performance of IPMC by optimizing these components.

## Optimization Strategies in IPMC Actuation

### Preparation Steps

Generally, the current state-of-the-art IPMC manufacturing techniques incorporate four steps: pretreatment (surface roughening), the initial compositing process [typically, impregnation-reduction (IR) and reductant permeation (RP)], the surface electroding process (typically, physical deposition and electroplating), and ion exchange. Each step involves many factors that affect IPMC performance, so optimization of the key factors is necessary (Takenaka et al., 1982; Millet et al., 1995; Kim and Kim, 2008; Hamburg et al., 2016). In 2012, Chang et al. proposed an effective method for preparing Pd-type IPMC (Chang et al., 2012). The overall preparation process was divided into three crucial steps, namely pretreatment, impregnation-reduction plating (IRP), and autocatalytic plating (ACP), and several specific procedures were focused on and improved, including the addition of a reducing agent and the time consumed. In terms of IPMC's own physical properties, its surface resistivity, bending stiffness, and dielectric coefficient will exert an influence its performance. In 2014, Wang et al. studied the physical and electromechanical properties of IPMC actuators and the effects of processing parameters on that performance (Wang et al., 2014c). They fabricated several IPMC samples with different features by combining certain crucial processing steps such as pretreatment, impregnation-reduction, and chemical plating. The formation mechanism of the surface and interface electrode in the processing steps was revealed, together with the effects of physical parameters on the performance of IPMC. Pretreatment provided a rough landscape, which increased the depth of electrode penetration and decreased the bending stiffness. The impregnation-reduction process mainly forms a granular penetration electrode between the mid-layer and the electrode layer. Due to the broad dispersion zone of penetrated electrode nanoparticles, the double-layer capacitance of IPMC was improved. Compared to a sample without roughening and chemical plating, the IPMC sample after roughening and chemical plating showed better performance, displaying a maximum of 10 mm under 1.7 V DC.

Furthermore, in 2016, Wang et al. performed a series of detailed experiments and revealed how the sandblasting method affected the surface and interfacial electrodes (Wang et al., 2016). Sandblasting is a convenient and controllable roughening method for treating the mid-layer without causing chemical damage. By changing the sandblasting time and powder size, a variety of surface profiles can be obtained on Nafion, and the corresponding parameters of the IPMC based on it, such as its surface resistance, equivalent modulus, and capacitance, can be optimized.

### Relaxation Elimination

When a DC voltage is applied to IPMC using water as a solvent, there is a corresponding rapid increase in deformation and then its slow reversal even beyond the initial position, which is called the relaxation effect of IPMC. The presence of relaxation will bring serious instability to IPMC actuators, which is unfavorable for the application of IPMC actuators in soft robotics. Currently, two methods have been developed to overcome the relaxation of IPMC: adjusting the water content and using an ionic liquid as a solvent (Bennett and Leo, 2004).

The water content of IPMC is a key factor affecting the relaxation. Wang et al. revealed the effects of dehydration on the electromechanical parameters of Au-type and Pd-type IPMCs (Wang et al., 2014b). The morphological evolution of surface electrodes depends on the water content of IPMC. Moreover, the physical parameters of IPMC, such as its stiffness, surface resistance, and capacitance, that are related to water content play an important role in the actuation process, which provides a way of understanding the effects of water content on the deformation mechanism of IPMC. The maximum deformation of IPMC without relaxation can be obtained by encapsulating IPMC at fixed water content (W3) (Barramba et al., 2007).

### Output Force Enhancement

Traditionally, Nafion 117 has been the most-used base membrane of IPMC, the thickness of which is around 180 μm. Due to the thinness of Nafion 117, the output force of the IPMC is lower than 10 mN, which will restrict IPMC use where a large output force is needed. Apparently, an effective way to improve the output force is to fabricate IPMC with a thicker base membrane. (Kim and Shahinpoor, 2002) fabricated IPMC with a thicker membrane through solution casting. An IPMC actuator with 1.12-mm thickness, 4-mm width, and 30-mm length can output 4.5 gf under 3 V DC. In 2011, He et al. studied the performance of IPMCs with various thicknesses (He et al., 2011). Under 3.5 V, with thickness increases from 0.22 to 0.42 mm and 0.80 mm, the deformation decreased by 26.1 and 47.3%, respectively, and the output force increased by 100.6 and 13.8%, respectively. The results indicated that as the thickness of the Nafion membrane increased, the output force of the IPMC was enhanced.

Another feasible method to improve the output force of IPMC is by doping additives into the polymer membrane (Oh and Jung, 2008; Yip et al., 2012). By doping water-soluble sulfonated multi-walled carbon nanotubes (sMWCNT) into the Nafion matrix, Ru et al. developed a new kind of IPMC actuator that has higher bending deformation and output force under low driving voltage; the output force can reach 14.37 mN when 3 V DC is applied (Ru et al., 2016). Wang et al. fabricated polymer membrane with different additives by solution casting, and analyzed the respective effects of doping with ethylene glycol (EG), dimethyl sulfoxide (DMSO), N,N0-dimethyl formamide (DMF), and N-methyl formamide (NMF) (Wang et al., 2014a). The EG-based IPMC actuator had better electromechanical properties at a 2 V DC voltage, with 1.5 mN output force and 4.0 mm tip deformation. Additionally, Nemat-Nasser et al. tried to improve the output force by altering the base membrane and movable cations (Nemat-Nasser et al., 2006). Furthermore, Ma et al. prepared IPMC with various additives to optimize the output force of IPMC (Ma et al., 2009).

Besides the methods mentioned above, some novel structures have been designed to increase the output force (Konyo et al., 2004; Zolfagharian et al., 2016). In 2018, Chang et al. developed IPMC actuators with single-layered electrodes, which led to distinct S-shaped deformation with large displacement and high output force (Chang L. et al., 2018).

### Effects of Interfacial Electrodes on IPMC Actuation

IPMC consists of an ionomer layer with metal electrode layers on either side. Between the ionomer layer and the surface electrode layer, there is an interfacial layer, which plays an important role in the performance of the IPMC. In particular, it has been clearly shown that the actuation capability of IPMC depends strongly on a high interfacial surface area between the metal and polymer because capacitance is directly related to the actuation performance of IPMC (Wang et al., 2017).

So far, the interfacial electrode of IPMC has been classified into two types, namely, dendritic and granular electrodes. Asaka et al. developed a structure of dendritic interfacial electrodes based on Au-type IPMC (Fujiwara et al., 2000). Chen et al. and Kim et al., respectively, proposed platinum and palladium granular interfacial structures (Shahinpoor and Kim, 2002). Their capacitance values are 1.5, 0.22, and 0.34 mF/cm^2^, respectively. The capacitance of the gold dendritic interfacial electrodes is 7 and 4 times as high as that of other structures. With an increase in the capacitance of interfacial surface electrodes, the electromechanical properties of IPMC can be effectively improved.

Recently, several methods have aimed at realizing the formation of a high interface surface area, including impregnation-reduction (IR), reductant permeation (RP), solution casting, and a direct assembly process. By combining the impregnation electroplating step (IEP) with the impregnation-reduction (IR) step, dendritic interfacial electrodes (DIEs) of palladium, platinum, silver, and copper inside an ionomer for IPMC can be obtained (Noh et al., 2002; Kim and Shahinpoor, 2003). DIEs are conducive to improvements in performance in IPMC actuation by reducing the metal consumption and increasing the surface area. In the initial research, DIEs were only formed by repeated IR with an Au complex.

Wang et al. proposed a rapid, facile, and efficient method to achieve the formation of Pd, Pt, Ag, and Cu DIEs inside the polymer membrane (Wang et al., 2017). Taking the Pd DIEs as an example, the main preparation steps are as follows. Firstly, the surface of the Nafion membrane should be roughened, which will increase the surface area and is favorable for the IR process of a Pd complex. The IR step is then performed after the palladium ion exchanging process. The reduction reaction occurs near the inner surfaces of the membrane, and the palladium atoms will grow along the thickness direction. The final step is to electroplate the membrane-containing palladium complex. As the reaction goes on, the palladium particles gradually grow up to become the main branches, then secondary branches are generated, and so on. The maximum depth of Pd DIEs can almost reach through to contact from either side of the ionic polymer. Because of the DIEs, the actuation performance of IPMC is effectively improved. This enhancement in the actuating performance of IPMC means that it will work better for actuators in soft robotics.

### IPMC as a Sensor

After external stimulus is exerted on IPMC, an electrical response will be generated according to the ion migration mechanism inside the IPMC (Bahramzadeh and Shahinpoor, 2011). When IPMC is used as a sensor in soft robotics, it can realize the perception of deformation, velocity, pressure, humidity, and even location-sensing through the distributed IPMC structure. Recently, many researchers have devoted themselves to researching and optimizing its sensing performance. Yamakita et al. proposed a system with IPMC sensor/actuator integration (Yamakita, 2006). Shahinpoor et al. reported the output voltage at 2 mV of an IPMC cantilever (40 × 5 mm) under 1-mm bending deformation (Shahinpoor et al., 1998). Konyo et al. developed an IPMC cantilever as a velocity sensor with a sensitivity of 9–12 mV/(m/s) (Konyo et al., 2004).

**Figure 3:**
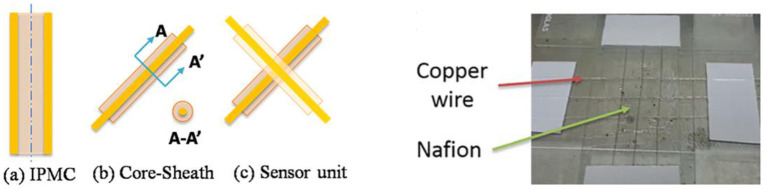
Conceptual design of an ionic polymer pressure sensor derived from the IPMC structure (Zhu et al., 2016a). In (b), the core is a single electrode wire, and the sheath is a Nafion membrane. (a) IPMC, (b) core-sheath, and (c) sensor unit **(Left)**. Image of the 3 × 3 ionic sensor network **(Right)** (reprinted with permission).

The main deficiency that restricts IPMC use as sensors is its low electrical response. To increase the output voltage, Zhu et al. proposed a novel IPMC sensor design with a 3 × 3 array that was achieved by solution casting (Figure 3) (Zhu et al., 2016a). Two different voltage responses were generated when pressure was applied to the IPMC array. Due to this design with a symmetrical structure, the output voltage reached up to 25 mV.

**Figure 4:**
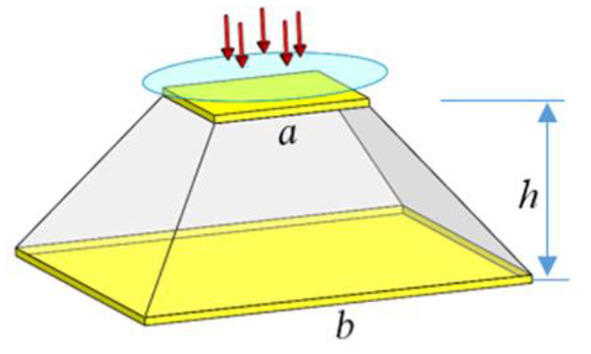
IPMC pressure sensor with a gradient shape (Zhu et al., 2019) [reproduced from Zhu et al. (2019) with the permission of AIP Publishing].

Subsequently, Zhu et al. studied the influence of the ambient humidity on Au-type IPMC under various humidity environments (Zhu et al., 2016b). According to the results, the response was seriously affected by humidity. In contrast to traditional strip-shaped IPMC sensors, Lei et al. presented a novel tube-shaped IPMC sensor that can respond to all stimuli perpendicular to the tube axis (Hong et al., 2016). Recently, Zhu et al. proposed a new ionic polymer pressure sensor with a gradient shape, fabricated by the casting method (Figure 4). The maximum response was much higher than that of the traditional strip-shaped IPMC (Zhu et al., 2019).

Additionally, Zhu et al. investigated the effects of different cations on the electrical responses of IPMC sensors at different ambient humidities (Zhu et al., 2016c). Volpini et al. established a predictive model to explain the dynamic compression-sensing response (Volpini et al., 2017). In 2017, Gudarzi designed an IPMC dynamic pressure sensor based on the streaming potential hypothesis and then presented charge current-generation mechanisms that operate due to the external pressure in both compression and shear modes (Gudarzi et al., 2017a,b). Considering the effect of the dimensions of the IPMC sensors, Wang et al. estimated the sensing performance of IPMC with various thicknesses, widths, and lengths (Wang et al., 2019).

## Conclusions and Remarks

As a soft transducer, IPMC has great potential in soft robotics, with the advantages of low weight, softness, flexibility, low power consumption, and self-sensing capacity. There are still some deficiencies, such as in its output force, relaxation, etc. that limit the application of IPMC in soft robotics.

Among the preparation steps of IPMC, there are still some pretreatment problems that need to be solved. Currently, the pretreatment methods of the base membrane mainly include sandpapering, chemical corrosion, plasma etching, and sandblasting. The membrane surface obtained by sandpapering is non-uniform and can be affected by human factors. Although chemical corrosion and plasma etching can result in a uniform surface, the molecular structure of the membrane surface is easy to break. Meanwhile, the surface uniformity of the base membrane is also hard to control with sandblasting. Therefore, a novel method to roughen the Nafion membrane still needs to be investigated to create a more uniform surface. The existing methods for eliminating relaxation also still have some disadvantages. Although using ionic liquids as the solvent can relieve relaxation, the response speed will decrease. Controlling the water content seems to be a better way to eliminate relaxation, but even a little variation in water content will lead to a large difference in performance. Additionally, difficulties arise with IPMC encapsulation at a specific water content. On the one hand, with a decrease in water content, the response speed will drop. On the other hand, due to the added encapsulation layer, the actuation of the IPMC will be weakened. Therefore, a compromise balance relation between relaxation elimination and maximizing performance needs to be achieved. To enhance the output force of IPMC, increasing the thickness of IPMC is a convenient approach. There are two ways to make the base membrane thicker: multilayer integration by hot pressing and solution casting. However, multilayers are easy to separate by long-term use, while casting a thick membrane will decrease the deformation and response rate. To improve the response rate of IPMC, Zhu et al. employed a high voltage pulse as the electric excitation signal for a short time in the early stage, by which the deformation attenuation of the thick IPMC could be avoided (Zhu et al., 2018).

In short, the existing solutions from our group have only partially relieved the deficiencies of IPMC, and these problems are not completely solved. Considering the favorable properties of IPMC, actuators and sensors in soft robotics made of this material will have promising prospects in the near future, and much research still needs to be done for its wide application.

## Author Contributions

MH: thoughts, writing–original draft. YW, ZZ, QH, DZ, and ML: discussion and investigation. YW: project administration. YW and ZZ: writing–review and editing.

### Conflict of Interest

The authors declare that the research was conducted in the absence of any commercial or financial relationships that could be construed as a potential conflict of interest.
